# Analysis of the *Pantoea ananatis* pan-genome reveals factors underlying its ability to colonize and interact with plant, insect and vertebrate hosts

**DOI:** 10.1186/1471-2164-15-404

**Published:** 2014-05-27

**Authors:** Pieter De Maayer, Wai Yin Chan, Enrico Rubagotti, Stephanus N Venter, Ian K Toth, Paul R J Birch, Teresa A Coutinho

**Affiliations:** Centre for Microbial Ecology and Genomics, University of Pretoria, Pretoria, 0002 South Africa; Department of Microbiology and Plant Pathology and Forestry and Agricultural Biotechnology Institute, University of Pretoria, Pretoria, 0002 South Africa; Genomics Research Institute, University of Pretoria, Pretoria, 0002 South Africa; James Hutton Institute, Cell and Molecular Sciences, Invergowrie, Dundee DD2 5DA UK; Division of Plant Science, University of Dundee (at JHI), Invergowrie, Dundee DD2 5DA UK

## Abstract

**Background:**

*Pantoea ananatis* is found in a wide range of natural environments, including water, soil, as part of the epi- and endophytic flora of various plant hosts, and in the insect gut. Some strains have proven effective as biological control agents and plant-growth promoters, while other strains have been implicated in diseases of a broad range of plant hosts and humans. By analysing the pan-genome of eight sequenced *P. ananatis* strains isolated from different sources we identified factors potentially underlying its ability to colonize and interact with hosts in both the plant and animal Kingdoms.

**Results:**

The pan-genome of the eight compared *P. ananatis* strains consisted of a core genome comprised of 3,876 protein coding sequences (CDSs) and a sizeable accessory genome consisting of 1,690 CDSs. We estimate that ~106 unique CDSs would be added to the pan-genome with each additional *P. ananatis* genome sequenced in the future. The accessory fraction is derived mainly from integrated prophages and codes mostly for proteins of unknown function. Comparison of the translated CDSs on the *P. ananatis* pan-genome with the proteins encoded on all sequenced bacterial genomes currently available revealed that *P. ananatis* carries a number of CDSs with orthologs restricted to bacteria associated with distinct hosts, namely plant**-**, animal**-** and insect**-**associated bacteria. These CDSs encode proteins with putative roles in transport and metabolism of carbohydrate and amino acid substrates, adherence to host tissues, protection against plant and animal defense mechanisms and the biosynthesis of potential pathogenicity determinants including insecticidal peptides, phytotoxins and type VI secretion system effectors.

**Conclusions:**

*P. ananatis* has an ‘open’ pan-genome typical of bacterial species that colonize several different environments. The pan-genome incorporates a large number of genes encoding proteins that may enable *P. ananatis* to colonize, persist in and potentially cause disease symptoms in a wide range of plant and animal hosts.

**Electronic supplementary material:**

The online version of this article (doi: 10.1186/1471-2164-15-404) contains supplementary material, which is available to authorized users.

## Background

*Pantoea ananatis* is a member of the family *Enterobacteriaceae* and is characterised by its ubiquity in nature and its frequent association with both plant and animal hosts. It has been found in a wide array of environments including rivers, soil samples, refrigerated beef and aviation fuel tanks [1, 2]. *P. ananatis* is most frequently isolated from plant materials, including roots, leaves and stems of a broad range of plant hosts and exists as part of the epiphytic and endophytic flora [1]. Since its identification as the causal agent of fruitlet rot of pineapple in the Philippines in 1928 [3], *P. ananatis* has been implicated in diseases of a wide range of host crops including maize and onion, *Eucalyptus*, sudangrass and honeydew melons [1, 4]. Individual isolates also appear to be capable of causing disease symptoms on a wide range of hosts. For example, strains pathogenic on rice and pineapple were demonstrated to cause blight symptoms on onion [4]. Conversely, some *P. ananatis* strains have been shown to promote plant growth [5, 6]. *P. ananatis* strains have also been found associated with insects, including tobacco thrips that act as vectors of onion-pathogenic strains, mulberry pyralids, ticks and fleas, demonstrating its ability to persist in invertebrate hosts [7–9]. Its implication in human infections reveals its capacity for proliferation and potential to cause disease in a vertebrate host [10, 11]. The ubiquity of *P. ananatis* suggests that it has adapted to proliferate in a wide range of environments, and its isolation from both plant and animal hosts indicate it has adapted for cross-Kingdom colonization and pathogenesis.

The concept of the pan-genome was introduced in 2005 [12]. The pan-genome of a bacterial species can be defined as the global gene repertoire of the species, and consists of a core genome, representing the genes present in all strains of the species, and an accessory genome composed of genes that are unique to particular strains as well as those genes that are absent from one or more of the sequenced strains [12, 13]. Core genes encode proteins that are generally involved in crucial cellular processes and are thus mostly vertically transferred from parent to progeny. Accessory genes, on the other hand, are prone to lateral gene transfer, and often encode functions related to niche adaptation [12–14]. The microbial pan-genome of a species can further be considered as ‘open’ or ‘closed’. A ‘closed’ pan-genome is highly conserved, and is typical associated with bacterial species which live in select niches, where they are secluded from the overall microbial gene pool or have a diminished capacity to acquire genes, such as *Bacillus anthracis* and *Mycobacterium tuberculosis* [13, 14]. By contrast an ‘open’ pan-genome is observed for bacterial species that can colonize and exploit several different environmental niches and can expand their accessory and pan-genome through different means of lateral gene transfer [12–14]. Four complete and four draft genomes of *P. ananatis* strains, isolated from various environmental sources, and with diverse lifestyles, have recently been sequenced. Here we have determined and characterized the open *P. ananatis* pan-genome and show its adaptive capacity to interact with hosts of both the animal and plant Kingdoms.

## Results and discussion

### *P. ananatis* genome statistics

**Figure 1:**
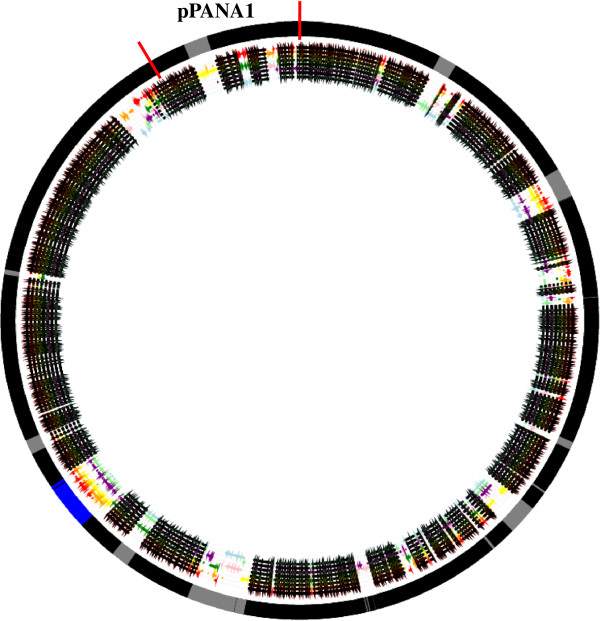
**Comparison of the CDS sets of eight sequenced** ***P. ananatis*** **strains.** The diagram was constructed using GenomeDiagram [70] on the basis of RBBH BlastP comparison of the CDS sets of each of the sequenced *P. ananatis* strains against the pan-genome CDS set. The strain order, from outside to inside, is AJ13355 (red), B1-9 (orange), BD442 (yellow), LMG20103 (green), LMG2665^T^ (purple), LMG5342 (purple), PA13 (light green) and PA4 (light blue). Core CDSs are colored as black arrows, while accessory CDS are colored in relation to the strain. pPANA1 plasmid CDS are demarcated by red lines. The outer ring shows the location of phage proteins (grey) and the ICE element (blue) in the pan-genome. The pPANA1 CDSs are demarcated by red lines.

The genomes of eight *P. ananatis* strains, isolated from different geographic locations and sources were sequenced [6, 15–18]. These include four complete and four high quality draft genome sequences (Table 1). The genomes consist of a chromosome, 4.39-4.61 Mb in size with an average G + C content of 53.7%, together with a large plasmid pPANA1, which is 281–353 kb in size and has an average G + C content of ~52% (Table 1). An estimated total of 4,225-4,415 CDSs are encoded on the combined chromosome and pPANA1 plasmid of each of the strains. Reciprocal Best BlastP Hit (RBBH) analysis [19] was used to undertake pair-wise comparisons of the protein complements encoded on the genomes of the eight strains, using the orthology cut-off values of >70% sequence identity and >70% sequence coverage between the query and hit [20]. This analysis indicated that between 89.3 and 95.7% of the proteins encoded on the genome of one strain have orthologs encoded on the genomes of the other strains, with an average amino acid identity of 99.4% (Figure 1, Additional file 1: Tables S1 and Additional file 2: Tables S2). These results suggested that an extensive, highly-conserved core genome, encompassing the majority of proteins encoded on each individual genome, exists among *P. ananatis* strains. Similar values were observed for *E. amylovora*, where on average 89% of the mean 3,819 CDSs predicted for each of twelve genomes compared are core, with an average amino acid identity of >99% between the core CDSs, while by contrast only 46.7% of the CDSs are core among three commensal and fourteen pathogenic strains of *Escherichia coli* (Table 2) [21, 22]. Alignment of the combined chromosome and pPANA1 nucleotide sequences also showed that extensive synteny exists among the four complete *P. ananatis* genomes (Additional file 3: Figure S1). Pairwise comparison of the *P. ananatis* protein sets against the total proteins encoded on the genome of the biological control strain *P. vagans* C9-1 [23], further indicated that an extensive set of core proteins may be conserved among different species within the genus *Pantoea*. Between 73.1-74% of the proteins encoded on each of the eight *P. ananatis* genomes shared orthology with proteins encoded on the genome of *P. vagans* C9-1, with an average amino acid identity of 84.8% (Additional file 1: Tables S1 and Additional file 2: Tables S2).

**Table 1:** **General genome properties of the eight** ***P. ananatis*** **strains**

Strain	Sequence status	Source	Lifestyle	Chromosome			Plasmid			Ref
				Size (Mb) G + C% #CDS			Size (Kb) G + C% #CDS			
AJ13355	Complete	Soil	Saprophyte	4.56	53.8	4,019	322	53.4	272	[15]
LMG20103	Complete	*Eucalyptus*	Pathogen	4.39	53.7	3,955	317	52.1	270	[16]
LMG5342	Complete	Human	Clinical isolate	4.61	53.5	4,098	303	51.5	255	[17]
PA13	Complete	Rice	Pathogen	4.59	53.7	4,156	281	52.3	238	[18]
LMG2665^T^	Draft	Pineapple	Pathogen	4.48^a^	53.5	4,043	306	51.8	259	-
PA4	Draft	Onion	Pathogen	4.57^a^	53.7	4,152	313	52.2	263	-
BD442	Draft	Maize	Pathogen	4.45^a^	53.6	4,049	353	51.1	320	-
B1-9	Draft	Onion	Plant-growth promoter	4.51^a^	53.7	4,074	314	51.7	267	[9]

The isolation source and predicted biological roles for each strain are shown. The *P. ananatis* genome consists of a chromosome and a large plasmid (LPP-1). The sizes, G + C contents (%) and number of proteins encoded on each replicon are indicated. ^a^indicates the genomes for which genome sizes were estimated based on the sum of contig lengths.

**Table 2:** **Pan-genome statistics derived from analyses of several bacterial taxa displaying ‘open’ pan-genomes**

Species	# strains	Mean # genome CDSs	Mean # core CDSs	% core CDSs/genome	Pan-genome (# CDS) compared strains	Estimated core genome (# CDS)	Unique CDSs per additional strain	Ref
*Streptococcus pneumoniae* ^*^	44	-	-	-	3,221	1,666^a^	1^a^	[24]
*Streptococcus agalactiae*	8	2,029	2,245	90.4%	>2,700	1,806	33	[12]
*Haemophilus influenzae* ^*^	13	1,793	1,461	81.5%	2,786	1,461	40	[25]
*Erwinia amylovora*	12	3,819	3,414	89.4%	5,751	-	52	[21]
*Pantoea ananatis*	8	4,342	4,033	92.9%	5,566	3,876	106	-
*Escherichia coli*	17	5,020	2,344	46.7%	>13,000	2,200	300	[22]
*Bacillus thuringiensis*	7	6,847	-	> 81%	>12,000	4,196	558	[26]

The number of strains included in the comparison are indicated, as are the mean number of CDSs per compared genome, those CDSs which are core to all compared strains, the relative proportion of core CDSs per genome (%) and the pan-genome sizes (#CDSs) for the compared strains, where the data is available. The estimated core genomes and unique CDSs added to the pan-genome when additional sequenced strains would be added to the comparison are shown. ^*^denotes those pan-genome analyses where the orthologous CDSs in a compared genome were first grouped ito orthologous clusters. ^a^indicates the number of unique genes that are predicted to be added to the *S. pneumoniae* pan-genome if 100 genomes would be compared.

### *P. ananatis* has an ‘open’ pan-genome

The pan-genome for the eight sequenced *P. ananatis* strains was determined by BlastP comparison of the translated CDS set, the clustering of orthologous proteins, and addition of the representatives of each orthologs cluster and strain-unique CDSs to the total pan-genome. The combined pan-genome for the eight compared *P. ananatis* strains encompasses 5,566 CDSs. Of these, 3,876 (69.6% of total CDSs) are core to all compared genomes (Figure 1). This implies that on average 89.5% of CDSs encoded on the genomes of each of the eight strains form part of the core genome determined for the eight compared strains. A total of 1,690 CDSs proteins (30.36% of the pan-genome total) make up the accessory fraction (Figure 1), of which an average of 108 CDSs are unique to each genome among the eight compared strains.

**Figure 2:**
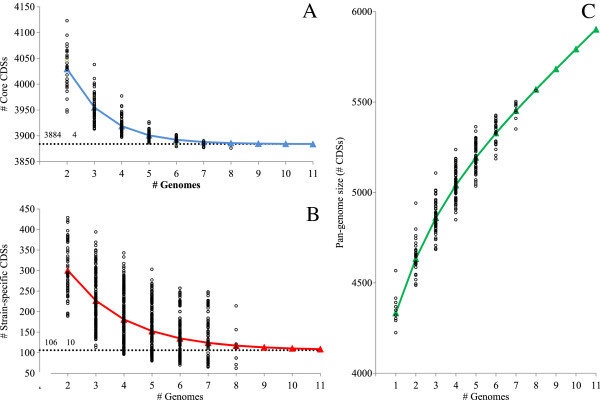
**Plots of the core CDSs, strain-specific CDSs and pan-genome CDSs of the species** ***P. ananatis*** . Comparisons of n = 1→8 genomes were performed to determine the core genome CDSs for the eight sequenced *P. ananatis* strains **(A)**, the strain-unique CDSs (TCs) added by each genome in the comparison **(B)**, and the *P. ananatis* pan-genome size (expressed as number of CDSs) **(C)**. Black circles indicate the data points. The data were fitted to a generalized least-squares non-linear model as per [12] to determine the core CDSs **(A)** and strain-unique CDSs **(B)** for the species when more sequenced strains are added to the comparison, as depicted by the triangles and trend line (blue for core CDSs and red for strain-unique CDSs) in the plots, while the dashed line shows the asymptotic core **(A)** and strain-specific CDSs **(B)** values for the species as predicted using the least-squares non-linear model function. The pan-genome for the species was extrapolated by fitting the strain-specific CDS data and average number of CDSs per compared genome to an algebraic function as per [12], indicated by green triangles **(C)**.

The sequential addition and BlastP comparison of the CDS sets of each of the eight *P. ananatis* strains, and BlastP comparison of all possible combinations as a function of the number of genomes *n* (where *n* = 2→8), was used to determine the number of core CDSs, the total pan-genome CDSs, and the strain-unique CDSs for each of the combinations. The resultant values were fitted to an exponential decay function [12] (Figure 2). By extrapolation of this function, the core, pan-genome and strain-unique CDSs for the entire species, beyond the scope of the eight sequenced strains, could be predicted [12]. On the basis of the asymptotic value from the exponential decay function, the estimated core genome for the species consists of 3,884 ± 4 CDSs (Figure 2A). This estimate is similar to the observed value for the comparison of the eight strains, indicating that the addition of more sequenced strains to the comparison will likely have a minimal effect on the core CDS fraction of the species. An estimated 106 ± 10 unique CDSs would be added to the species pan-genome if a new sequenced genome is incorporated into the comparison (Figure 2B). A rarefaction curve plotted on the basis of the average number of CDSs per sequenced genome and the estimated strain-unique CDSs (for *n* = 2→8) shows that the extrapolated curve continues to increase with the addition of new sequenced genomes to the analysis (Figure 2C). This typifies an ‘open’ pan-genome as has been observed in similar pan-genome analyses for a number of bacterial species, members of which can inhabit a wide range of environments and/or have diverse lifestyles and possess efficient means of lateral gene transfer [12–14, 21, 22, 24–26] (Table 2). By contrast, similar rarefaction curves reach an asymptotic value of zero within a limited pan-genomic context in species exhibiting a ‘closed’ pan-genome [12]. For example, no new genes are accumulated in the pan-genome of *Bacillus anthracis* when a fourth genome is added to the comparison, which may be linked to the more isolated niche this pathogen occupies [12, 27]. The ‘open’ pan-genome of *P. ananatis* may thus reflect the diverse habitats from which strains of this species have been isolated and their different lifestyles. Similar pan-genome analyses in *E. coli* revealed that ~300 CDSs would be added to the pan-genome with each novel genome sequenced, while 52 unique CDSs would be added with each sequenced genome of the phytopathogen *E. amylovora* [21, 22] (Table 2). Thus the *P. ananatis* pan-genome can be considered to be less ‘open’ than that of *E. coli*, but more ‘open’ than that of *E. amylovora*. This assumption must, however, be viewed with caution, as several additional factors may influence the unique CDS and core- and pan-genome calculations, such as genome completeness and annotation, the number of strains compared, strains selection, genome size, as well as the orthology cut-off values and methodologies employed [12–14, 28].

### The *P. ananatis* accessory genome encodes mainly ‘poorly characterized’ proteins

**Figure 3:**
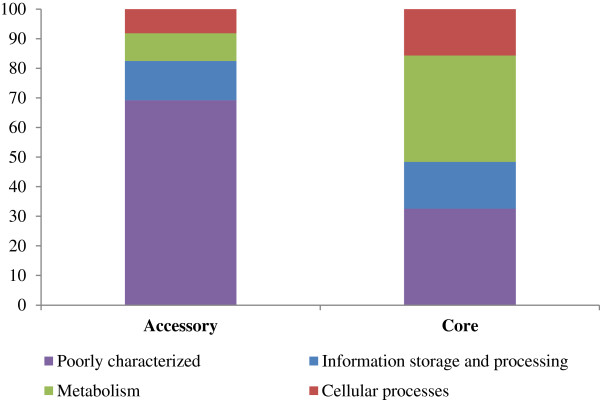
**Relative proportions (%) of core and accessory CDSs in each COG super-functional category.** The COG functional and super-functional categories for each of the *P. ananatis* pan-genome CDSs were determined by submitting the amino acid sequences to the COGnitor server and searching against the COG database [29].

The translated protein products of the CDSs encoded in both the accessory and core fractions of the *P. ananatis* pan-genome were classified into super-functional and functional categories, on the basis of orthology to functionally characterized proteins as determined using the COGnitor tool and according to the classification nomenclature employed in the Conserved Orthologous Groups Database [29] (Additional file 4: Table S3). The majority of the 1,690 proteins encoded on the accessory genome (69.2%) fall into the super-functional COG category of ‘poorly characterized’ proteins, while relatively small proportions of proteins encoded on the accessory genome belong to the metabolism (9.4%) and cellular processes (8.2%) super-functional categories (Figure 3; Additional file 4: Table S3). This finding is supported by analyses of the *E. coli* and *Staphylococcus aureus* pan-genomes, where the majority of proteins encoded on the accessory genome also fell into the ‘poorly characterized’ super-functional category [13]. By contrast, a large proportion of the 3,876 proteins encoded on the *P. ananatis* core genome are involved in the super-function metabolism (35.94%), with only 32.6% belonging to the ‘poorly characterized’ super-functional category (Figure 3; Additional file 4: Table S3). Similar proportions of core and accessory proteins are involved in information storage and processing. Within this super-functional category, however, most of the proteins encoded on the core genome are involved in transcription, translation, ribosomal structure and biogenesis, while the majority of accessory genome-encoded proteins are involved in DNA replication, recombination and repair (Additional file 4: Table S3). This super-functional category includes transposases, integrases and other mobile genetic elements, and their extensive representation in the accessory genome indicates that horizontal gene transfer has potentially played a significant role in the diversification of the eight *P. ananatis* strains.

### Prophage integration and integrative and conjugative elements have played a major role in the diversification of *P. ananatis* strains

Integrated bacteriophage elements, or prophages, were identified in the genome sequences of the eight *P. ananatis* strains using Prophinder [30]. Between two and four prophages are integrated into the replicons of each of the eight strains, and 699 accessory CDSs (41.4% of the total accessory CDSs) are encoded by these prophages, indicating that phage integration has played a substantial role in *P. ananatis* diversification. Between 24.2% and 74.3% of the strain-unique CDSs for each of the eight strains are localized in predicted integrated phage elements (Figure 1). This suggests that a large fraction of the strain-unique CDS complement of any new sequenced strains would likely be derived from phages. Prophages are found in two-thirds of all γ-proteobacteria and have been shown to play a major role in bacterial evolution through the horizontal transfer of genetic factors that contribute to various processes within the bacterial host, including fitness and pathogenesis [31]. Examples of prophage-borne genes in other bacteria include those encoding a Shiga toxin in *E. coli*, Type III secretion system effectors in *S. enterica*, R- and F-type bacteriocins in *Pseudomonas aeruginosa*, and a superoxide dismutase (providing protection against oxidative defences within the mammalian host) in *S. enterica* [32–35]. The prevalence of prophage CDSs among the accessory portion of the pan-genome, suggest that they may also play a major role in the adaptive evolution of this species, potentially contributing to their ability to colonize various environmental niches and hosts.

A further 148 CDSs (8.7% of the total accessory CDSs) are encoded in integrative and conjugative elements (ICEs), which are present in the genomes of five of the sequenced strains (Figure 1). These ICEs, in other bacterial taxa, including *S. enterica* and *Vibrio cholerae*, have been shown to disseminate and confer a number of adaptive traits, including antibiotic and heavy metal resistance [36].

### The *P. ananatis* pan-genome encodes a number of proteins found on the genomes of bacteria associated with distinct hosts

**Figure 4:**
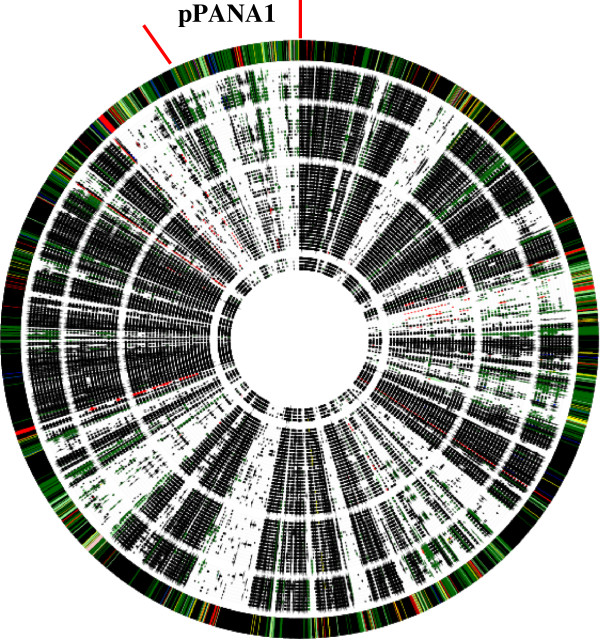
**Comparative diagram of the** ***P. ananatis*** **pan-genome against members of the genomes of representative members of the family** ***Enterobacteriaceae*** **showing that AAB-, IAB- and PAB-specific CDSs are mainly restricted to regions of lower conservation among the** ***Enterobacteriaceae*** . The *P. ananatis* pan-genome CDS set was compared by RBBH approach against the CDS sets of 54 members of the family *Enterobacteriaceae*, which are listed in Additional file 7: Table S6. Orthologs were considered as those translated CDSs sharing >50% amino acid identity and >70% sequence coverage across the query and hit sequences. Orthologs in each of the enterobacterial comparators are indicated by black arrows The outer circle shows the locations of PAB- (dark green), non-enterobacterial PAB- (light green), IAB- (maroon), AAB- (red), *Pantoea-* (blue) and *P. ananatis*-specific (yellow) CDSs on the *P. ananatis* pan-genome.

As *P. ananatis* strains are frequently found associated with a wide range of different hosts including plants, insects and humans, the *P. ananatis* pan-genome was analyzed to identify CDSs that may be involved in host-microbe interactions. The translated protein products of the 5,566 pan-genome CDSs were compared by BlastP comparison against the NCBI non-redundant (nr) protein database [37, 38]. This revealed a large number of *P. ananatis* proteins with orthology to proteins encoded on the genomes of microorganisms associated with distinct hosts, namely animal-associated bacteria (AAB), plant-associated bacteria (PAB) and insect-associated (IAB) bacteria. A total of 1,415 CDSs (25.4% of the total pan-genome CDSs) shared orthology with CDSs specific to microorganisms belonging to one group (i.e. specific to the AAB, IAB or PAB groups but not more than one group). Figure 4 depicts a comparison of the *P. ananatis* pan-genome translated CDS products against the protein sets of 54 members of the *Enterobacteriaceae* for which complete genomes are available. This diagram showed the occurrence of AAB, IAB and PAB-specific CDSs mainly within large regions of reduced conservation among the *Enterobacteriaceae*, indicating they have likely arisen through extensive horizontal acquisition events. The BlastP analysis also identified a number of CDSs that were either unique to *P. ananatis* (106 CDSs -1.9% of total pan-genome) or specific to the *Pantoea* genus (28 CDSs - 0.5% of total pan-genome CDSs). While pan-genome CDSs with orthologs in the genomes of both plant and animal-associated bacteria are almost certain to play a role in the interactions with hosts in both Kingdoms, the large number of PAB-, AAB-, and IAB-specific CDSs in the *P. ananatis* pan-genome may provide mechanisms specific to these different Kingdoms and were investigated further.

### The *P. ananatis* pan-genome encodes proteins that may be involved in colonization of animal hosts

A total of 151 *P. ananatis* CDSs (2.7% of total pan-genome CDSs) encode proteins with orthologs restricted to animal-associated bacteria (AAB), including animal-pathogenic *Salmonella, Escherichia* and *Yersinia* strains, with between 61 (LMG5342) and 84 (AJ13355) AAB-specific CDSs encoded on the different *P. ananatis* genomes. Of these, 50 CDSs form part of the *P. ananatis* core genome, while 101 are found in the accessory portion of the pan-genome, suggesting possible adaptive evolution of some strains that has enabled colonization of and persistence in animal hosts. The majority of proteins encoded by the AAB-specific CDSs (110 CDSs – 66.3% of total AAB-specific CDSs) belong to the ‘poorly characterized’ super-functional category. However, a small proportion is involved in metabolism (21 CDSs) and cellular processes (17 CDSs) (Additional file 5: Table S4). Two sets of CDSs encode proteins putatively involved in the transport and metabolism of the carbohydrate substrates glucuronide and mannose, respectively. Orthologs of several *P. ananatis* pan-genome CDSs with a potential role in attachment, and in defense against antimicrobials are also restricted in distribution to AAB (Additional file 5: Table S4). Two CDSs unique among the sequenced *P. ananatis* strains to AJ13355, encode proteins with a predicted role in the biogenesis of a type 1 fimbria, with orthologs restricted to *E. coli* and *Salmonella* spp. A putative non-fimbrial autotransporter adhesin, which is common to all sequenced *P. ananatis* strains, shared extensive sequence identity with the AidA-I and MisL adhesins of enteropathogenic *E. coli* and *S. enterica*, respectively. These adhesins are involved in intestinal adherence in these pathogens [39, 40]. Orthologs of the *S. enterica* Mig-14 protein, which has been proposed to repress immune system functions [41], are also present in all strains. Similarly, the protein products of two *P. ananatis* pan-genome CDSs showed orthology to β-lactamases and their cognate transcriptional regulators in a number of AAB, including clinical strains of *Enterobacter cloacae* (Sfo-1/AmpR) and *Citrobacter sedlakii* (Sed1/SedR). These provide resistance to a broad spectrum of antibiotics used in the clinical environment [42, 43], and given their isolation from this environment [10, 11], may form the basis for antibiotic resistance of clinical *P. ananatis* strains.

Analysis of the *P. ananatis* pan-genome has thus revealed the presence of various CDSs coding for proteins with potential roles in adherence, immunity suppression, antibiotic-resistance and carbohydrate metabolism proteins that may aid in the persistence of this species in animal hosts (Additional file 5: Table S4).

### The *P. ananatis* pan-genome encodes proteins with a potential role in interactions with insect hosts

Fifteen pan-genome CDSs (0.3% of total pan-genome CDSs) share orthologs only in insect-associated bacterial genera, including *Photorhabdus* and *Wolbachia*. Between two (AJ13355 and PA13) and thirteen (LMG20103) of these IAB-specific CDSs are present in each of the individual *P. ananatis* genomes. In particular, one locus found in the genomes of four *P. ananatis* strains encodes twelve proteins showing sequence homology to a locus in *Photorhabdus luminescens* subsp. *laumondii* TTO1 and several *Streptomyces* spp. (Additional file 5: Table S4). Within this locus PANGEN_3511 and PANGEN_3515 encoded orthologs of two nikkomycin biosynthetic proteins in *P. luminescens* (plu1441 and plu1874). This antibiotic is produced by *Streptomyces* spp. and has acaricidal, fungicidal and insecticidal activities [44]. PANGEN_3520 and PANGEN_3522 encoded orthologs of the *Streptomyces rubellomurinus* FrbC and FrbD proteins involved in production of an antimalarial compound (ABB90932-90933 - 50% average amino acid identity) [45]. *P. ananatis* strains may thus have acquired a locus for the biosynthesis of a potential insecticidal peptide. As *P. ananatis* is frequently isolated from invertebrate hosts, the presence of such a peptide may be of interest for the biological control of insect pests [7–9].

### The *P. ananatis* pan-genome encodes proteins with a potential role in plant-microbe interactions

A large number of the *P. ananatis* pan-genome CDSs (1,249 CDSs – 22.4% of total pan-genome content) shared orthology with CDSs restricted to plant-associated bacteria, and between 933 (PA4) and 985 (LMG2665^T^) of these are encoded on each individual *P. ananatis* genome. This finding concurs with the frequent isolation of *P. ananatis* from the plant environment [1]. Of these CDSs, 849 formed part of the pan-genome core, while 400 were associated with the accessory genome. While the majority of PAB-specific CDSs were found in common with both enterobacterial and non-enterobacterial plant-associated species, a relatively large number (200 CDSs – 16% of total PAB-specific CDSs) shared orthology only with CDSs restricted to non-enterobacterial PAB. This suggests that extensive horizontal exchange has occurred between *P. ananatis* and non-enterobacterial PAB. A total of 200 PAB-specific CDSs are localized on the pPANA1 pan-plasmid (50.5% of the total pan-plasmid CDSs). Previously we showed that the pPANA1 plasmid is part of the Large *Pantoea* Plasmid group (LPP-1) common to all sequenced *Pantoea* spp. The LPP-1 plasmids share a small set of common core CDSs and a much larger accessory component, and we postulated that they play a major role in the ecological diversification of the genus [46, 47]. The large number of PAB-specific CDSs (Figure 4) indicates that the pPANA1 plasmid likely plays a major role in the adaptation of *P. ananatis* to colonize and interact with plants.

As is the case for AAB-specific CDSs, the largest proportion of PAB-specific CDSs encoded proteins that belong to the ‘poorly characterized’ super-functional category (46.2% of the total PAB-specific CDSs), but a substantial number of PAB-specific CDSs encode proteins involved in metabolism (30.4%) and other cellular processes (11.5%). These may play a role in efficient colonization, nutrient utilization and persistence in or on the plant and/or in other plant-microbe interactions. An extensive set of PAB-specific CDSs encode proteins with a role in the transport and metabolism of carbohydrates (Additional file 6: Table S5), which may facilitate the uptake and metabolism of plant-derived carbohydrates. A number of orthologs of proteins involved in the degradation of plant carbohydrates are also encoded by PAB-specific CDSs present in all *P. ananatis* strains, including a predicted endo-1,4-β-xylanase, two polygalacturonases, a putative pectin acetylesterase, as well as a predicted cellulase with extensive sequence identity to the minor cellulose Cel8Y of *Dickeya dadantii* [48] (Additional file 6: Table S5). Several PAB-restricted amino acid transport and metabolism systems are also encoded on the *P. ananatis* pan-genome, including orthologs of the opine octopine, released from plant tumors induced by *Agrobacterium tumefaciens* [49] and the Amadori compound deoxyfructosyl glutamine, found in rotting fruits and vegetables, and in tumors caused by chrysopine-type *Agrobacterium* strains [50] (Additional file 6: Table S5). These compounds could furthermore serve as carbon, nitrogen and energy sources for *P. ananatis* in the plant. Several PAB-specific proteins with a predicted role in iron uptake and metabolism were also found to be encoded on the *P. ananatis* pan-genome. These included a predicted hydroxamate siderophore transporter, siderophore receptor, ferric dicitrate sensor components FecI/FecR and a PAB-specific TonB-ExbBD complex for outer membrane iron transport (Additional file 6: Table S5). These PAB-specific proteins may allow *P. ananatis* to actively contest for the limited iron available in the plant environment [51].

Several CDSs encoding PAB-restricted proteins potentially involved in protection against plant defenses are present in the *P. ananatis* pan-genome. PANGEN_04534-4535 encode orthologs of the protein Ohr and its transcriptional regulator OhrR, which are involved in resistance to organic hydroperoxides produced by plants in defense to pathogen infection [52]. Several PAB-restricted multidrug efflux pumps that could play a role in the extrusion of plant-produced antimicrobials and β-lactamases, which may specifically degrade plant antimicrobials, are encoded on the *P. ananatis* pan-genome (Additional file 6: Table S5). PANGEN_05563 encodes an ortholog of the cyclic β-1,2-glucan polysaccharide biosynthetic protein NdvB. This polysaccharide has been shown to provide protection to *Xanthomonas campestris* against localized and systemic defenses in the host plant [53]. A PAB-specific locus on the pPANA1 plasmid of all eight *P. ananatis* strains encodes orthologs of the proteins BudABCR, which are involved in the production of 2,3-butanediol. This volatile has been shown to promote plant growth and induce systemic resistance in the plant host [54, 55], which may be linked to the biological role as plant growth promoter ascribed to strains of this species [5, 6].

### The *P. ananatis* pan-genome encodes proteins with potential roles in plant- and animal-pathogenesis

As strains of *P. ananatis* have been found to be pathogenic on a broad range of plant hosts as well as humans, the pan-genome was analyzed to identify potential molecular determinants underlying its pathogenicity. Our analyses revealed the absence of many of the factors that are central to the pathogenicity and virulence arsenal of related plant and animal pathogens, including animal toxins, hemolysins, phytotoxins, several secretion systems (Type II, III and IV) and their associated effectors. However, several genes and loci with orthology to characterized pathogenicity determinants in related animal and plant pathogens could be identified in the *P. ananatis* pan-genome.

**Figure 5:**
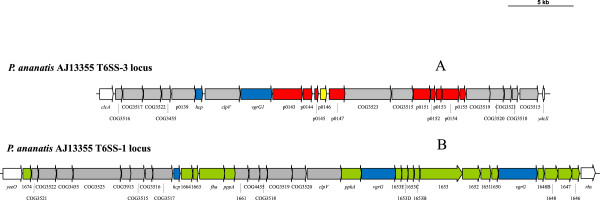
**The T6SS-1 and T6SS-3 loci of** ***P. ananatis*** **encode proteins with orthologs restricted to plant-associated and animal-associated bacteria.** The T6SS-3 **(A)** and T6SS-1 **(B)** loci of *P. ananatis* AJ13355 are shown as an example. T6SS core genes with orthologs in both PAB and AAB groups are colored in grey, while PAB- and AAB-specific genes are colored in green and red, respectively. The *hcp* and *vgrG* genes are denoted by blue arrows, while the yellow arrow represents a *P. ananatis*-specific gene.

Type VI secretion systems (T6SSs) have been identified in a number of human pathogenic bacteria, including *P. aeruginosa* and *V. cholerae* [56, 57], as well as plant pathogens such as *Pseudomonas syringae* and *Pectobacterium atrosepticum* [58, 59]. The T6SS serves as injectisome for effector proteins including the hemolysin co-regulated protein (Hcp) and valine-glycine repeat (VgrG) protein [56–59]. Previously we have described the presence of three Type VI secretion system loci in *P. ananatis*, namely T6SS-1, -2 and -3 [60]. The T6SS-1 and -2 loci are found on the genomes of all *P. ananatis* strains, while T6SS-3 is restricted to the pPANA1 plasmids of *P. ananatis* AJ13355, LMG20103 and PA4. Non-conserved islands are localized adjacent to the *vgrG* and *hcp* genes in the T6SS-1 and T6SS-3 loci. On the basis of the presence of conserved domains and structural homology to various proteins of known function, we postulated that the proteins encoded in these islands may be translocated across the outer membrane or into the host cell cytosol as terminal domains of the VgrG and Hcp effectors, and that they may play a role in various functions in a wide range of hosts, including animal and plant pathogenesis [60]. During the classification of the *P. ananatis* pan-genome CDSs as AAB-, IAB- or PAB-specific we observed that the proteins encoded in the *vgrG* island of the T6SS-3 locus shared orthology only with proteins encoded on the genomes of members of the AAB group. A further non-conserved island encoding proteins with orthologs specific to AAB was found in the T6SS-3 locus (Figure 5A; Additional file 5: Table S4). While the orthologs of most of these T6SS-3 island proteins were annotated as hypothetical proteins and belong to the ‘poorly characterized’ super-functional COG category, their localization within the T6SS putative effector islands together with the fact that they are AAB-group specific, makes them interesting targets for further analysis. Similarly, PAB-specific proteins were found to be encoded by CDSs localized within the *vgrG* and *hcp* islands of the T6SS-1 locus (Figure 5B; Additional file 6: Table S5). This suggests a potential role for this secretion system in the translocation of effectors into the plant host. Other PAB-specific proteins in the T6SS-1 locus included a serine/threonine protein kinase (PpkA) and phosphatase (PppA) and a FHA domain protein, which have been shown to play a role in the post-translational regulation of the Type VI secretion system [61]. A further partial Type VI secretion system (T6SS-2) was also identified in all *Pantoea* and *Erwinia* species [60]. All proteins encoded in this locus are restricted to PAB, suggesting a potential role for T6SS-2 in plant-microbe interactions.

Analysis of the PAB-specific complement also showed the presence of a CDS encoding an ice-nucleation protein with orthologs restricted to plant-pathogenic strains of *P. ananatis*, *Pantoea agglomerans*, *X. campestris* and *P.s syringae*. This protein induces wounds in frost-damaged plants and is postulated to allow these pathogens to gain access to host tissues [62]. A locus on the pPANA1 plasmids of *P. ananatis* LMG2665^T^ and B1-9 encodes proteins (PANGEN_05466-5474) with orthology to proteins for the biosynthesis of non-ribosomal peptide synthases/polyketide synthases (NRPS/PKS). The potential function of this locus in NRP/PK biosynthesis was further substantiated by comparison of the locus against the antiSMASH server [63]. Many phytopathogenic bacteria have been shown to carry CDSs encoding NRPS/PKS that are required for the production of phytotoxins, e.g. *P. syringae* syringomycin and syringopeptin, and *P. atrosepticum* coronofacic acid conjugates [64]. Orthologs of the proteins encoded in the *P. ananatis* NRPS/PKS locus were found to be restricted to PAB (*E. amylovora* ATCC BAA-2158 - EAIL5_2884-2892; 72% average amino acid identity) and may thus potentially encode synthases for the production of a phytotoxin. However, the biological role of the NRPS/PKS products in *P. ananatis* will need to be determined.

## Conclusions

*Pantoea ananatis* is ubiquitous in the environment and has an inherent capacity to survive, proliferate and form intimate relationships with plants, as well as insect and human hosts [1]. In particular, its frequent isolation from both plant and animal hosts suggests it has adapted to colonize, proliferate and potentially cause disease in these hosts. Here we analyzed the genome sequences of eight *P. ananatis* strains. As has been observed for other members of the *Enterobacteriaceae*, *P. ananatis* exhibits an ‘open’ pan-genome, which is mainly influenced by the integration of phage elements, but also by integrative conjugative elements and other insertion elements. Phages play a significant role in bacterial evolution, transferring fitness and pathogenicity factors to their bacterial host [30]. They could therefore represent major adaptive drivers for *P. ananatis*, allowing strains of this species to colonize and interact with both plant and animal hosts.

Analysis of the *P. ananatis* pan-genome CDS complement revealed the presence of a large number of proteins restricted in distribution to plant- and animal-associated bacteria (PAB and AAB). These include a number of factors that could serve as putative tools for *P. ananatis* adherence and colonization of host tissues, to utilize nutrients and persist within a host(s), and potentially cause disease. However, it cannot be excluded that common mechanisms underlying colonization, persistence and pathogenicity exist among bacteria that are associated with both plant and animal hosts. The ability of a bacterium to make a cross-Kingdom jump is dependent on several prerequisites, including the close and frequent contact with the novel host, the ability to overcome host defences, and the capacity for horizontal acquisition of genes encoding factors that enable the bacterium to persist in its new host [65]. The frequent isolation of *P. ananatis* from various different environments and the (pan-)genomic evidence of a bacterial species well-adapted towards survival in and interaction with different hosts, provide a primary indication of the ecological success of *P. ananatis* and how it may have evolved to interact with cross-Kingdom hosts.

## Methods

### Genome comparisons and construction of the *P. ananatis* pan-genome

The genome sequences from eight *P. ananatis* strains, four complete and four partially assembled genomes (Table 1), were included in the study. The partial genomes were annotated using FgenesB [66]. CDS sets were standardized by local BlastN analysis to identify ORFs which may not have been predicted for a particular genome. The combined nucleotide sequences of the chromosome and pPANA1 plasmid, for the *P. ananatis* strains for which complete genomes were available, were aligned using Mauve v. 2.3.1. [67]. The translated CDS sets for each of the eight genomes were pair-wise compared by local BlastP analysis using Bioedit 7.1.11 software package [68]. The comparison was performed using the reciprocal best hit approach (RBBH), whereby orthologs were assumed when a Blast hit of a query protein in the compared subject protein set also returned the query protein sequence as the best Blast hit when it in turn was compared by BlastP analysis against the query strain protein set [19]. The number of orthologous CDSs and average amino acid identities (sum of the amino acid identities for each compared protein divided by the aligned length of the protein) for each combination of pair-wise compared proteins sets were determined. Orthologs were defined using cut-off values (>70% amino acid identity and >70% sequence coverage for the query and hit) [20]. Using these orthology parameters and localized BlastP comparison, the orthologous CDSs from each of the genomes were clustered [69], where each cluster represented the set of RBBH orthologs for each distinct CDS across all the compared genomes. A representative of each cluster, the longest member sequence of each cluster for CDSs shared by one or more strain, as well as CDSs unique to specific *P. ananatis* strains, were incorporated in a single pan-genome file. The translated protein products of the entire pan-genome CDS set were compared against the Conserved Orthologous Groups database using COGnitor [29] to determine the COG functional and super-functional category to which they belong. Prophages and phage proteins were identified by searching against the ACLAME database using Prophinder [30]. A diagram of the eight CDS sets compared against the pan-genome CDS set was constructed on the basis of the above localized BlastP analysis results using GenomeDiagram [70].

### Pan-genome calculations

The size of the pan-genome for the eight sequenced strains, as well as the core and accessory fractions were determined by localized BlastP analysis with the translated CDS sets of each of the sequence *P. ananatis* strain against the pan-genome CDS set. The core genome, signifying all pan-genome CDSs common to all eight strains, and the accessory genome, incorporating those CDSs which are absent in or more of the strains or were unique to the genome of a particular strain were tabulated, and the pan-genome determined as the sum of the core and accessory CDSs.

The sequential inclusion of the CDS sets of each of the eight genomes in all possible combinations was used to determine the core CDSs, accessory CDSs shared by more than one but not all strains, and those unique to a single strain, as a function of the number of genomes (*n*) in the comparison (where *n* = 1,2,…8). The estimated strain-specific CDSs and core CDSs for the species, beyond the scope of eight sequenced strains if the genomes of additional strains were sequenced (i.e. *n*→ ∞), were extrapolated by fitting the data for the *n* = 1→8 comparison combinations above to an exponential decay functions as per [12]. The data was fitted to the function using the generalized least squares (gnls) algorithm of the nlme (linear and non-linear mixed-effects models) package in R [71]. The estimated data points for *n*→∞ obtained from the function along with the actual data points from the comparison of the eight *P. ananatis* CDS sets were plotted in graphs (Figure 2A and B) of the number of genomes versus the number of core/strain-specific CDSs. In order to extrapolate the pan-genome size for the species, the strain-unique CDSs and average number of CDSs per compared genome (for *n* = 2→8 genomes compared) were incorporated into an algebraic formula as per [12].

### Comparison between the *P. ananatis* pan-genome and all available genomes

The CDSs encoded by *P. ananatis* were compared by BlastP analysis against the NCBI non-redundant (nr) protein database [38]. For this comparison, orthology was assumed for proteins sharing >30% amino acid identity and 70% sequence coverage between the query and hit [72]. On the basis of the Blast hits, CDSs were identified that shared orthologs in bacteria occupying distinct ecological niches, namely those associated with animals (AAB), insects (IAB) and plants (PAB), as determined from available information of their source of isolation. For the purpose of this grouping, only those bacteria that are specifically associated with animals and plant tissues and/or the rhizosphere environment were taken into consideration, while members that are frequently found associated with hosts of both Kingdoms, such as *Klebsiella* and *Enterobacter* sp., were disregarded.

A diagram of the comparison between the translated protein products encoded by the *P. ananatis* pan-genome CDS set and the protein sets of 54 members of the *Enterobacteriaceae*, encompassing all the genera for which complete genomes were available (Additional file 7: Table S6), was constructed using GenomeDiagram [70], using the RBBH approach for localized BlastP comparison. Orthologs were considered when proteins shared >50% amino acid identity and >70% coverage between the hit and query sequences.

### Availability of supporting data

The complete genome sequences of *P. ananatis* LMG20103 (chromosome + plasmid: NC_013956.2), AJ13355 (chromosome: NC_017531.1; plasmid: NC_017533.1), PA13 (chromosome: NC_017554.1; plasmid: NC_017553.1) and LMG5342 (chromosome: NC_016816.1; plasmid: NC_016817.1), as well as the draft genomes of *P. ananatis* B1-9 (CAEI00000000 and CAEJ00000000), LMG2665T ((JMJJ00000000), PA4 (JMJK00000000) and BD442 (JMJL00000000) are publically available on the NCBI database under the given accession numbers. The protein datasets for all eight *P. ananatis* strains, as well as the R scripts, input and output files for the pan-genome calculations and pan-genome graphs are available in the LabArchives repository [73].

## Electronic supplementary material

Additional file 1: Table S1: Number and proportion (%) of protein coding sequences (CDSs) shared between pair-wise compared *P. ananatis* strains. Values are given for the strains in each row in relation to the comparator strains in the top row. The CDS sets of each of the eight *P. ananatis* strains were also compared to *P. vagans* C9-1 [23]. (DOCX 13 KB)

Additional file 2: Table S2: Average amino acid identities (%) of the translated proteins products for shared CDSs between pair-wise compared *P. ananatis* strains. Values are given for the strains in each row in relation to the comparator strains in the top row. The CDS sets of each of the eight *P. ananatis* strains were also compared to the *P. vagans* C9-1 CDS set [23]. (DOCX 12 KB)

Additional file 3: Figure S1: Genome alignment showing extensive synteny between *P. ananatis* genomes. The nucleotide sequences of the combined chromosome and plasmid replicons of the four *P. ananatis* strains for which complete genome sequences are available were aligned using Mauve 2.3.1 [67]. The mauve and magenta blocks indicate two syntenic blocks in the chromosomes, while the blue block denotes the aligned pPANA1 plasmids. (PPTX 89 KB)

Additional file 4: Table S3: COG classification of the *P. ananatis* pan-genome core and accessory CDSs. The number and proportion (%) of core and accessory CDSs in each COG functional and super-functional (in bold) category are shown. (DOCX 14 KB)

Additional file 5: Table S4: *P. ananatis* AAB- and IAB-specific CDSs. The closest relatives carrying orthologs, their locus tags and percentage amino acid identity are shown. The latter was calculated by dividing the sum of amino acid identities for translated CDSs in a locus, by the total lengths of the aligned protein sequences. Putative functions for the AAB- and IAB-specific CDSs are shown. The presence of the AAB-specific CDSs or loci in the different *P. ananatis* strains (1. AJ13355, 2. LMG5342, 3. LMG20103, 4. PA13, 5. BD442, 6. PA4, 7. B1-9 and 8. LMG2665^T^), are indicated by a ‘+’, while absence is denoted by ‘-‘. AAB-specific CDSs are colored in red, while IAB-specific CDSs are in maroon. (XLSX 10 KB)

Additional file 6: Table S5: *P. ananatis* PAB-specific CDSs. The closest relatives carrying orthologs, their locus tags and percentage amino acid identity are shown. The latter was calculated by dividing the sum of amino acid identities for CDSs in a locus, by the total lengths of the aligned protein sequences. Putative functions for the PAB-specific CDSs are shown. The presence of the PAB-specific CDSs in the different *P. ananatis* strains (1. AJ13355, 2. LMG5342, 3. LMG20103, 4. PA13, 5. BD442, 6. PA4, 7. B1-9 and 8. LMG2665^T^), are indicated by a ‘+’, while absence is denoted by ‘-‘. PAB-specific CDSs are colored in dark green, while NEPAB-specific CDSs are in light green. (XLSX 19 KB)

Additional file 7: Table S6: List of *Enterobacteriaceae* used for the construction of the pan-genome comparative diagram. The different *Enterobacteriaceae* to which the *P. ananatis* pan-genome CDS complement were compared are shown, as are the Genbank accession numbers for each of the comparator strains. (XLSX 12 KB)

## References

[1] Coutinho TA, Venter SN (2009). *Pantoea ananatis*: an unconventional plant pathogen. Mol Plant Pathol.

[2] Ercolini D, Russo F, Torrieri E, Masi P, Villani F (2006). Changes in the spoilage-related microbiota of beef during refrigerated storage under different packaging conditions. Appl Environ Microbiol.

[3] Serrano FB (1928). Bacterial fruitlet brown-rot of pineapple in the Philippines. Philip J Sci.

[4] Kido K, Hasegawa M, Hioyuki M, Kobayashi M, Yuichi T (2010). *Pantoea ananatis* strains are differentiated into three groups based on reactions of tobacco and welsh onion and on genetic characteristics. J Gen Plant Pathol.

[5] Kim WI, Cho WK, Kim SN, Chu H, Ryu KY, Yun JC, Park CS (2011). Genetic diversity of cultivable plant growth-promoting rhizobacteria in Korea. J Microbiol Biotechnol.

[6] Kim HJ, Lee JH, Kang BR, Rong X, McSpadden Gardener BB, Ji HJ, Park CS, Kim YC (2012). Draft genome sequence of *Pantoea ananatis* B1-9, a nonpathogenic plant growth-promoting bacterium. J Bacteriol.

[7] Wells ML, Gitaitis RD, Sanders FH (2002). Association of tobacco thrips, *Frankliniella fusca* (Thysanoptera: thripidae), with two species of bacteria of the genus *Pantoea*. Ann Entomol Soc Am.

[8] Watanabe K, Sato M (1999). Gut colonization of an ice nucleation active bacterium, *Erwinia* (*Pantoea*) *ananas*, reduces the cold hardiness of mulberry pyralid larvae. Cryobiology.

[9] Murrell A, Dobson SJ, Yang X, Lacey E, Barker SC (2003). A survey of bacterial diversity in ticks, lice and fleas from Australia. Parasitol Res.

[10] Brenner DJ, Fanning GR, Leete Knutson JK, Steigerwalt AG, Krichevsky MI (1984). Attempts to classify herbicola group-*Enterobacter agglomerans* strains by deoxyribonucleic acid hybridization and phenotypic tests. Int J Syst Bacteriol.

[11] De Baere T, Verhelst R, Labit C, Verschraegen G, Wauters G, Claeys G, Vaneechoutte M (2004). Bacteremic infection with *Pantoea ananatis*. J Clin Microbiol.

[12] Tettelin H, Masignani V, Cieslewicz MJ, Donati C, Medini D, Ward NL, Angiuoli SV, Crabtree J, Jones AL, Durkin AS, DeBoy RT, Davidsen TM, Mora M, Scarselli M, Margarit y R I, Peterson JD, Hauser CR, Sundaram JP, Nelson WC, Madupu R, Brinkac LM, Dodson RJ, Rosovitz MJ, Sullivan SA, Daugherty SC, Haft DH, Selengut J, Gwinn ML, Zhou L, Zafar N (2005). Genome analysis of multiple pathogenic isolates of *Streptococcus agalactiae*: implications for the microbial “pan-genome”. Proc Natl Acad Sci U S A.

[13] Mira A, Martín-Cuadrado AB, D’Auria G, Rodríguez-Valera F (2010). The bacterial pan-genome: a new paradigm in microbiology. Int Microbiol.

[14] Medini D, Donati C, Tettelin H, Masignani V, Rappuoli R (2005). The microbial pan-genome. Curr Opin Genet Dev.

[15] Hara Y, Kadotani N, Izui H, Katashkina JI, Kuvaeva TM, Andreeva IG, Golubeva LI, Malko DB, Makeev VJ, Mashko SV, Kozlov YI (2012). The complete genome sequence of *Pantoea ananatis* AJ13355, an organism with great biotechnological potential. Appl Microbiol Biotechnol.

[16] De Maayer P, Chan WY, Venter SN, Toth IK, Birch PR, Joubert F, Coutinho TA (2010). Genome sequence of *Pantoea ananatis* LMG20103, the causative agent of *Eucalyptus* blight and dieback. J Bacteriol.

[17] De Maayer P, Chan WY, Rezzonico F, Bühlmann A, Venter SN, Blom J, Goesmann A, Frey JE, Smiths TH, Duffy B, Coutinho TA (2012). Complete genome sequence of clinical isolate *Pantoea ananatis* LMG5342. J Bacteriol.

[18] Choi O, Lim JY, Seo YS, Hwang I, Kim J (2012). Complete genome sequence of the rice pathogen *Pantoea ananatis* strain PA13. J Bacteriol.

[19] Moreno-Hagelsieb G, Latimer K (2008). Choosing BLAST options for better detection of orthologs as reciprocal best hits. Bioinformatics.

[20] Halachev MR, Loman NJ, Pallen MJ (2012). Calculating orthologs in bacteria and Archaea: a divide and conquer approach. PLoS One.

[21] Mann RA, Smiths THM, Bühlmann A, Blom J, Goesmann A, Frey JE, Plummer KM, Beer SV, Luck J, Duffy B, Rodoni B (2013). Comparative genomics of 12 strains of *Erwinia amylovora* identifies a pan-genome with a large conserved core. PLoS One.

[22] Rasko DA, Rosovitz MJ, Myers GS, Mongodin EF, Fricke WF, Gajer P, Crabtree J, Sebaihia M, Thomson NR, Chaudhuri R, Henderson IR, Sperandio V, Ravel J (2008). The pangenome structure of *Escherichia coli*: comparative genomic analysis of *E. coli* commensal and pathogenic isolates. J Bacteriol.

[23] Smits THM, Rezzonico F, Kamber T, Goesmann A, Ishimaru CA, Stockwell VO, Frey JE, Duffy B (2010). Genome sequence of the biocontrol agent *Pantoea vagans* C9-1. J Bacteriol.

[24] Donati C, Hiller NL, Tettelin H, Muzzi A, Croucher NJ, Angiuoli SV, Oggioni M, Dunning Hotopp JC, Hu FZ, Riley DR, Covacchi A, Mitchell TJ, Bentley SD, Kilian M, Ehrlich GD, Rappuoli R, Moxon ER, Masignani V (2010). Structure and dynamics of the pan-genome of *Streptococcus pneumoniae* and closely related species. Genome Biol.

[25] Hogg JS, Hu FZ, Janto B, Boissy R, Hayes J, Keefe R, Post JC, Ehrlich GD (2007). Characterization and modeling of the *Haemophilus influenzae* core and supragenomes based on the complete genomic sequences of Rd and 12 clinical nontypable strains. Genome Biol.

[26] Fang Y, Li Z, Liu J, Shu C, Wang X, Zhang X, Yu X, Zhao D, Liu G, Hu S, Zhang J, Al-Mssallem I, Yu J (2011). A pangenomic study of *Bacillus thuringiensis*. J Genet Genomics.

[27] Fraser-Liggett CM (2005). Insights on biology and evolution from microbial genome sequencing. Genome Res.

[28] Tettelin H, Riley D, Cattuto C, Medini D (2008). Comparative genomics: the bacterial pan-genome. Curr Opin Microbiol.

[29] Tatusov RL, Galperin MY, Natale DA, Koonin EV (2000). The COG database: a tool for genome-scale analysis of protein functions and evolution. Nucl Acid Res.

[30] Lima-Mendez G, Van Helden J, Toussaint A, Leplae R (2008). Prophinder: a computational tool for prophage prediction in prokaryotic genomes. Bioinformatics.

[31] Brüssow H, Canchaya C, Hardt W-F (2004). Phages and the evolution of bacterial pathogens: from genomic rearrangements to lysogenic conversion. Microbiol Mol Biol Rev.

[32] Plunkett G, Rose DJ, Durfee TJ, Blattner FR (1999). Sequence of Shiga toxin 2 phage 933 W from *Escherichia coli* O157:H7: Shiga toxin as a phage late-gene product. J Bacteriol.

[33] Mirold S, Rabsch W, Tschäpe H, Hardt WD (2001). Transfer of the *Salmonella* type III effector *sopE* between unrelated phage families. J Mol Biol.

[34] Nakayama K, Takashima K, Ishihara H, Shinomiya T, Kageyama M, Kanaya S, Ohnishi M, Murata T, Mori H, Hayashi T (2000). The R-type pyocin of *Pseudomonas aeruginosa* is related to P2 phage, and the F-type is related to lambda phage. Mol Microbiol.

[35] Figueroa-Bossi N, Uzzau S, Maloriol D, Bossi L (2001). Variable assortment of prophages provides a transferable repertoire of pathogenic determinants in *Salmonella*. Mol Microbiol.

[36] Wozniak R, Waldor M (2010). Integrative and conjugative elements: mosaic mobile genetic elements enabling dynamic lateral gene flow. Nat Rev Microbiol.

[37] Altschul SF, Gish W, Miller W, Myers EW, Lipman DJ (1990). Basic local alignment search tool. J Mol Biol.

[38] *National Centre for Biotechnology Information Protein Database*. http://www.ncbi.nlm.nih.gov/protein

[39] Dorsey CW, Laarakker MC, Humphries AD, Weening EH, Bäumler AJ (2005). *Salmonella enterica* serotype Typhimurium MisL is an intestinal colonization factor that binds fibronectin. Mol Microbiol.

[40] Henderson IR, Navarro-Garcia F, Desvaux M, Fernandez RC, Ala’Aldeen D (2004). Type V protein secretion pathway: the autotransporter story. Microbiol Mol Biol Rev.

[41] Valdivia RH, Cirillo DM, Lee AK, Bouley DM, Falkow S (2000). *mig-14* is a horizontally acquired, host-induced gene required for *Salmonella enterica* lethal infection in the murine model of typhoid fever. Infect Immun.

[42] Matsumoto Y, Inoue M (1999). Characterization of SFO-1, a plasmid-mediated inducible class A β-lactamase from *Enterobactercloacae*. Antimicrob Agents Chemother.

[43] Petrella S, Clermont D, Casin I, Jarlier V, Sougakoff W (2001). Novel class A β-lactamase Sed-1 from *Citrobacter sedlakii*: genetic diversity of β-lactamases within the *Citrobacter*genus. Antimicrob Agents Chemother.

[44] Liao G, Li J, Li L, Yang H, Tian Y, Tan H (2010). Cloning, reassembling and integration of the entire nikkomycin biosynthetic gene cluster into *Streptomyces ansochromogenes* lead to an improved nikkomycin production. Microb Cell Fact.

[45] Eliot AC, Griffin BM, Thomas PM, Johannes TW, Kelleher NL, Zhao H, Metcalf WW (2008). Cloning, expression, and biochemical characterization of *Streptomyces rubellomurinus* genes required for biosynthesis of the potent antimalarial compound FR900098. Chem Biol.

[46] De Maayer P, Chan WY, Blom J, Venter SN, Duffy B, Smits TH, Coutinho TA (2012). The large universal *Pantoea* plasmid LPP-1 plays a major role in biological and ecological diversification. BMC Genomics.

[47] Smits THM, Rezzonico F, Kamber T, Blom J, Goesmann A, Ishimaru CA, Frey JE, Stockwell VO, Duffy B (2011). Metabolic versatility and antibacterial metabolite biosynthesis are distinguishing genomic features of the fire blight antagonist *Pantoea vagans* C9-1. PLoS One.

[48] Boccara M, Aymeric J, Camus C (1994). Role of endoglucanases in *Erwinia chrysanthemi* 3937 virulence on *Saintpaulia ionantha*. J Bacteriol.

[49] Zanker H, Von Lintig J, Schröder J (1992). Opine transport genes in the octopine (*occ*) and nopaline (*noc*) catabolic regions in Ti plasmids of *Agrobacterium tumefaciens*. J Bacteriol.

[50] Baek CH, Farrand SK, Lee KE, Park DK, Lee JK, Kim KS (2003). Convergent evolution of Amadori opine catabolic systems in plasmids of *Agrobacterium tumefaciens*. J Bacteriol.

[51] Wandersman C, Delepelaire P (2004). Bacterial iron sources: from siderophores to hemophores. Annu Rev Microbiol.

[52] Mongkolsuk S, Praituan W, Loprasert S, Fuangthong M, Chamnongpol S (1998). Identification and characterization of a new organic hydroperoxide resistance (*ohr*) gene with a novel pattern of oxidative stress regulation from *Xanthomonas campestris* pv. phaseoli. J Bacteriol.

[53] Rigano LA, Payette C, Brouillard G, Marano MR, Abramowicz L, Torres PS, Yun M, Castagnaro AP, Oirdi ME, Dufour V, Malamud F, Dow JM, Bouarab K, Voijnov AA (2007). Bacterial cyclic β-(1,2)-glucan acts in systemic suppression of plant immune responses. Plant Cell.

[54] Ryu CM, Farag MA, Hu CH, Reddy MS, Wei HX, Paré PW, Kloepper JW (2003). Bacterial volatiles promote growth in *Arabidopsis*. Proc Natl Acad Sci U S A.

[55] Ryu CM, Farag MA, Hu CH, Reddy MS, Kloepper JW, Paré PW (2004). Bacterial volatiles induce systemic resistance in *Arabidopsis*. Plant Physiol.

[56] Mougous JD, Cuff ME, Raunser S, Shen A, Zhou M, Gifford CA, Goodman AL, Joachimiak G, Ordoñez CL, Lory S, Walz T, Joachimiak A, Mekalanos JJ (2006). A virulence locus of *Pseudomonas aeruginosa* encodes a protein secretion apparatus. Science.

[57] Pukatzki S, Ma AT, Sturtevant D, Krastins B, Sarracino D, Nelson WC, Heidelberg JF, Mekalanos JJ (2006). Identification of a conserved bacterial protein secretion system in *Vibrio cholerae* using the *Dictyostelium* host model system. Proc Natl Acad Sci U S A.

[58] Haapalainen M, Mosorin H, Dorati F, Wu RF, Roine R, Taira S, Nissinen R, Mattinen L, Jackson R, Pirhonen M, Lin HC (2012). Hcp2, a secreted protein of the phytopathogen *Pseudomonas syringae* pv. tomato DC3000, is required for fitness for competition against bacteria and yeasts. J Bacteriol.

[59] Liu H, Coulthurst SJ, Pritchard L, Hedley PE, Ravensdale M, Humphris S, Burr T, Takle G, Brurberg MB, Birch PR, Salmond GP, Toth IK (2008). Quroum sensing coordinates brute force and stealth modes of infection in the plant pathogen *Pectobacterium atrosepticum*. PLoS Pathog.

[60] De Maayer P, Venter SN, Kamber T, Duffy B, Coutinho TA, Smits THM (2011). Comparative genomics of the type VI secretion systems of *Pantoea* and *Erwinia* species reveals the presence of putative effector islands that may be translocated by the VgrG and Hcp proteins. BMC Genomics.

[61] Mougous JD, Gifford CA, Ramsdell TL, Mekalanos JJ (2007). Threonine phosphorylation post-translationally regulates protein secretion in *Pseudomonas aeruginosa*. Nat Cell Biol.

[62] Lindow SE, Arny DC, Upper CD (1982). Bacterial ice nucleation: a factor in frost injury to plants. Plant Physiol.

[63] Medema MH, Blin K, Cimermancic P, de Jager V, Zakrzweski P, Fischbach MA, Weber T, Takano E, Breitling R (2011). antiSMASH: rapid identification, annotation and analysis of secondary metabolite biosynthesis gene clusters in bacterial and fungal genome sequences. Nucl Acids Res.

[64] Donadio S, Monciardini P, Sosio M (2007). Polyketide syntases and nonribosomal peptide synthetases: the emerging view from bacterial genomics. Nat Prod Rep.

[65] van Baarlen P, van Belkum A, Summerbell RC, Crous PW, Thomma BPHJ (2007). Molecular mechanisms of pathogenicity: how do pathogenic microorganisms develop cross-kingdom host jumps?. FEMS Microbiol Rev.

[66] *Softberry FgenesB: bacterial operon and gene prediction server*. http://linux1.softberry.com/berry.phtmlhttp://linux1.softberry.com/berry.phtml

[67] Darling AC, Mau B, Blattner FR, Perna NT (2004). Mauve: multiple alignment of conserved genomic sequences with rearrangements. Genome Res.

[68] Hall TA (1999). BioEdit: a user-friendly biological sequence alignment editor and analysis program for Windows 95/98/NT. Nucl Acids Symp Ser.

[69] Kittichotirat W, Bumgarner RE, Asikainen S, Chen C (2011). Identification of the pangenome and its components in 14 distinct *Aggregatibacter actinomycetemcomitans* strains by comparative genomics analysis. PLoS One.

[70] Pritchard L, White JA, Birch PR, Toth IK (2006). GenomeDiagram: a python package for the visualization of large-scale genomic data. Bioinformatics.

[71] Ihaka R, Gentleman R (1996). R: A language for data analysis and graphics. J Comput Graphic Stat.

[72] Konstantinidis KT, Tiedje JM (2005). Towards a genome-based taxonomy for prokaryotes. J Bacteriol.

[73] *LabArchive repository*. https://mynotebook.labarchives.com/share/BMC_Genomics_Pan-genome_paper_data/Ni41fDM4MTI0LzUvVHJlZU5vZGUvMjQzMDQzNjg0NXwxNi41 (http://dx.doi.org/10.6070/H4PZ56S0)

