# Supplementary material for: Analysis of the Pantoea ananatis pan-genome reveals factors underlying its ability to colonize and interact with plant, insect and vertebrate hosts
Source: BMC Genomics. 2014 May 27;15(1):404. doi: 10.1186/1471-2164-15-404 (PMC4070556; doi:10.1186/1471-2164-15-404)
Supplement: Supplementary file 2 — Additional file 2: Table S2: Average amino acid identities (%) of the translated proteins products for shared CDSs between pair-wise compared P. ananatis strains. Values are given for the strains in each row in relation to the comparator strains in the top row. The CDS sets of each of the eight P. ananatis strains were also compared to the P. vagans C9-1 CDS set [23]. (DOCX 12 KB) [file 12864_2013_6141_MOESM2_ESM.docx]

**Supplementary Table S2 Average amino acid identities (%) of the translated proteins products for shared CDSs between pair-wise compared *P. ananatis* strains.**

| **Species** | **Strain** | AJ13355 | LMG20103 | LMG5342 | PA13 | BD442 | PA4 | LMG2665 | B1-9 | C9-1 |
| --- | --- | --- | --- | --- | --- | --- | --- | --- | --- | --- |
| *P. ananatis* | AJ13355 | 100 |  |  |  |  |  |  |  |  |
| *P. ananatis* | LMG20103 | 99.41 | 100 |  |  |  |  |  |  |  |
| *P. ananatis* | LMG5342 | 99.36 | 99.36 | 100 |  |  |  |  |  |  |
| *P. ananatis* | PA13 | 99.41 | 99.26 | 99.40 | 100 |  |  |  |  |  |
| *P. ananatis* | BD442 | 99.35 | 99.34 | 99.32 | 99.52 | 100 |  |  |  |  |
| *P. ananatis* | PA4 | 99.46 | 99.35 | 99.30 | 99.34 | 99.41 | 100 |  |  |  |
| *P. ananatis* | LMG2665 | 99.57 | 99.40 | 99.34 | 99.33 | 99.40 | 99.43 | 100 |  |  |
| *P. ananatis* | B1-9 | 99.46 | 99.38 | 99.26 | 99.43 | 99.36 | 99.52 | 99.58 | 100 |  |
| *P. vagans* | C9-1 | 84.82 | 84.67 | 84.75 | 84.95 | 85.07 | 84.91 | 84.57 | 84.74 | 100 |
