# Supplementary material for: Analysis of the Pantoea ananatis pan-genome reveals factors underlying its ability to colonize and interact with plant, insect and vertebrate hosts
Source: BMC Genomics. 2014 May 27;15(1):404. doi: 10.1186/1471-2164-15-404 (PMC4070556; doi:10.1186/1471-2164-15-404)
Supplement: Supplementary file 3 — Additional file 3: Figure S1: Genome alignment showing extensive synteny between P. ananatis genomes. The nucleotide sequences of the combined chromosome and plasmid replicons of the four P. ananatis strains for which complete genome sequences are available were aligned using Mauve 2.3.1 [67]. The mauve and magenta blocks indicate two syntenic blocks in the chromosomes, while the blue block denotes the aligned pPANA1 plasmids. (PPTX 89 KB) [file 12864_2013_6141_MOESM3_ESM.pptx]

## Slide 1
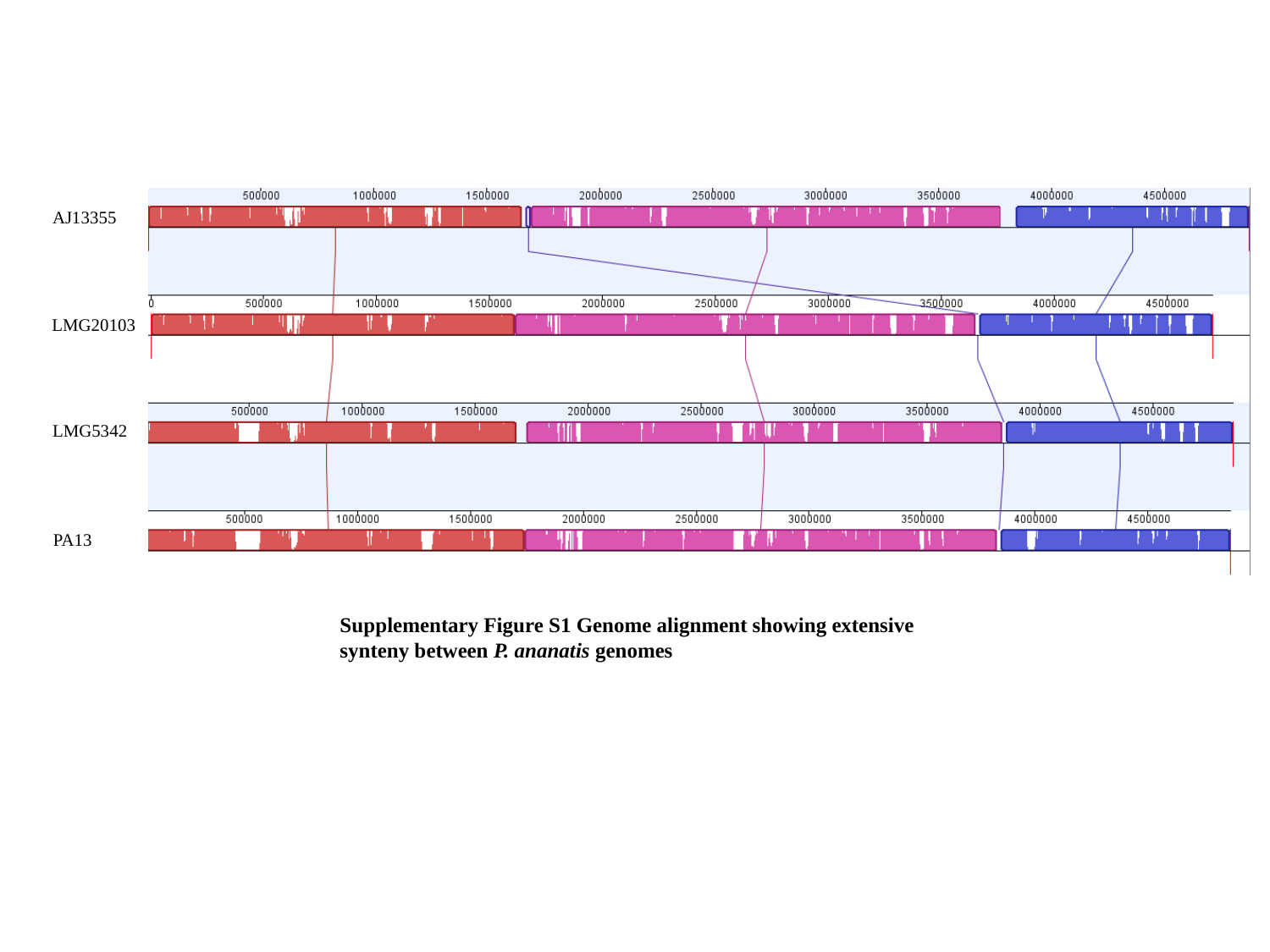

AJ13355
LMG20103
LMG5342
PA13
Supplementary Figure S1 Genome alignment showing extensive synteny between P. ananatis genomes
