# Supplementary material for: Analysis of the Pantoea ananatis pan-genome reveals factors underlying its ability to colonize and interact with plant, insect and vertebrate hosts
Source: BMC Genomics. 2014 May 27;15(1):404. doi: 10.1186/1471-2164-15-404 (PMC4070556; doi:10.1186/1471-2164-15-404)
Supplement: Supplementary file 4 — Additional file 4: Table S3: COG classification of the P. ananatis pan-genome core and accessory CDSs. The number and proportion (%) of core and accessory CDSs in each COG functional and super-functional (in bold) category are shown. (DOCX 14 KB) [file 12864_2013_6141_MOESM4_ESM.docx]

**Table S3 COG classification of the *P. ananatis* pan-genome core and accessory CDSs.**

|  | **Accessory** |  | **Core** |  |
| --- | --- | --- | --- | --- |
|  | # CDS | % Accessory | # CDS | % Core |
| **Information storage and processing** | **224** | **13.22** | **613** | **15.82** |
| DNA replication, recombination and repair | 155 | 9.15 | 130 | 3.35 |
| Transcription | 63 | 3.72 | 289 | 7.46 |
| Translation, ribosomal structure and biogenesis | 6 | 0.35 | 194 | 5.01 |
| **Cellular processes** | **138** | **8.15** | **608** | **15.69** |
| Cell division and chromosome partitioning | 5 | 0.30 | 31 | 0.80 |
| Cell motility and chemotaxis | 12 | 0.71 | 87 | 2.24 |
| Cell wall/membrane/envelope biogenesis | 59 | 3.48 | 183 | 4.72 |
| Intracellular secretion and protein trafficking | 27 | 1.59 | 56 | 1.44 |
| Posttranslational modification | 16 | 0.94 | 113 | 2.92 |
| Signal transduction | 19 | 1.12 | 110 | 2.84 |
| Defense mechanisms | 0 | 0.00 | 28 | 0.72 |
| **Metabolism** | **159** | **9.39** | **1393** | **35.94** |
| Amino acid transport and metabolism | 43 | 2.54 | 359 | 9.26 |
| Carbohydrate transport and metabolism | 37 | 2.18 | 322 | 8.31 |
| Coenzyme synthesis and metabolism | 11 | 0.65 | 128 | 3.30 |
| Energy production and conversion | 15 | 0.89 | 178 | 4.59 |
| Inorganic ion transport and metabolism | 14 | 0.83 | 202 | 5.21 |
| Lipid metabolism | 20 | 1.18 | 81 | 2.09 |
| Nucleotide transport and metabolism | 5 | 0.30 | 84 | 2.17 |
| Secondary metabolite biosynthesis | 14 | 0.83 | 39 | 1.01 |
| **Poorly characterized** | **1169** | **69.01** | **1262** | **32.56** |
| General function prediction only | 90 | 5.31 | 427 | 11.02 |
| Function unknown | 1079 | 63.70 | 835 | 21.54 |
| **Total CDS** | **1690** |  | **3876** |  |
